# Associations of mid-pregnancy HbA1c with gestational diabetes and risk of adverse pregnancy outcomes in high-risk Taiwanese women

**DOI:** 10.1371/journal.pone.0177563

**Published:** 2017-05-15

**Authors:** Yi-Ran Ho, Panchalli Wang, Mei-Chun Lu, Shih-Ting Tseng, Chun-Pai Yang, Yuan-Horng Yan

**Affiliations:** 1 Department of Endocrinology and Metabolism, Kuang Tien General Hospital, Taichung, Taiwan; 2 Department of Obstetrics and Gynecology, Ditmanson Medical Foundation Chia-Yi Christian Hospital, Chiayi, Taiwan; 3 Department of Medical Research, Kuang Tien General Hospital, Taichung, Taiwan; 4 Department of Food and Nutrition, Providence University, Taichung City, Taiwan; 5 Department of Neurology, Kuang Tien General Hospital, Taichung, Taiwan; 6 Department of Nutrition and Institute of Biomedical Nutrition, Hung Kuang University, Taichung, Taiwan; 7 Institute of Occupational Medicine and Industrial Hygiene, College of Public Health, National Taiwan University, Taipei, Taiwan; University of North Carolina at Chapel Hill, UNITED STATES

## Abstract

**Background:**

The objective of this study was to investigate the associations among the mid-pregnancy glycated hemoglobin A1c (HbA1c) level, gestational diabetes (GDM), and risk of adverse pregnancy outcomes in women without overt diabetes and with positive 50-g, 1-h glucose challenge test (GCT) results (140 mg/dL or greater).

**Methods:**

This prospective study enrolled 1,989 pregnant Taiwanese women. A two-step approach, including a 50-g, 1-h GCT and 100-g, 3-h oral glucose tolerance test (OGTT), was employed for the diagnosis of GDM at weeks 23–32. The mid-pregnancy HbA1c level was measured at the time the OGTT was performed. A receiver operating characteristic (ROC) curve was used to determine the relationship between the mid-pregnancy HbA1c level and GDM. Multiple logistic regression models were implemented to assess the relationships between the mid-pregnancy HbA1c level and adverse pregnancy outcomes.

**Results:**

An ROC curve demonstrated that the optimal mid-pregnancy HbA1c cut-off point to predict GDM, as diagnosed by the Carpenter-Coustan criteria using a two-step approach, was 5.7%. The area under the ROC curve of the mid-pregnancy HbA1c level for GDM was 0.70. Compared with the levels of 4.5–4.9%, higher mid-pregnancy HbA1c levels (5.0–5.4, 5.5–5.9, 6.0–6.4, 6.5–6.9, and >7.0%) were significantly associated with increased risks of gestational hypertension or preeclampsia, preterm delivery, admission to the neonatal intensive care unit, low birth weight, and macrosomia (the odds ratio [OR] ranges were 1.20–9.98, 1.31–5.16, 0.88–3.15, 0.89–4.10, and 2.22–27.86, respectively).

**Conclusions:**

The mid-pregnancy HbA1c level was associated with various adverse pregnancy outcomes in high-risk Taiwanese women. However, it lacked adequate sensitivity and specificity to replace the two-step approach in the diagnosis of GDM. The current study comprised a single-center prospective study; thus, additional, randomized control design studies are required.

## Introduction

For the previous 30 years, investigators have attempted to determine whether the glycated hemoglobin A1c (HbA1c) level during pregnancy may be used as a screening or diagnostic test for gestational diabetes (GDM) [1–3]. Technological advances have made HbA1c a more standardized and user-friendly test with broad availability; however, in general, previous studies have been consistent with previous unsuccessful attempts [4–6]. For pregnant women without diabetes, screening for potential GDM using a mid-pregnancy oral glucose tolerance test (OGTT) has been suggested. However, more recent studies have indicated that the HbA1c level during pregnancy may predict GDM in women at high risk for diabetes [7,8]. Furthermore, these studies predominately focused on early-pregnancy and first-trimester HbA1c levels, whereas the association between mid-pregnancy HbA1c levels and GDM was limited.

During pregnancy, the HbA1c level exhibits biphasic changes, with decreases between the first trimester and mid-pregnancy, followed by increases in the third trimester [9]. Based on this physiological phenomenon, the HbA1c level at the first trimester, mid-pregnancy and third trimester may represent different biomarkers with different cut-off points, which may be linked to GDM and adverse pregnant outcomes. Previous studies have indicated that in early pregnancy and the first-trimester HbA1c level elevations may represent a useful measurement to screen women with GDM. However, in the second-trimester and later pregnancy, the HbA1c level could not replace the OGTT for GDM diagnosis [10]. Moreover, a recent study indicated that the mid-pregnancy HbA1c level may potentially reduce the number of OGTTs [11]. The HbA1c level in mid-pregnancy was investigated as a predictor of diabetes following GDM [12]. The role of the mid-pregnancy HbA1c level in the diagnosis of GDM was not fully understood. Furthermore, there were several advantages of mid-pregnancy HbA1c compared with the earlier stage HbA1c, such as consistency with the time point of the OGTT, willingness of pregnant women to receive the test, and an optimal timing of intervention. Thus, additional studies regarding mid-pregnancy HbA1c were necessary.

The association between the HbA1c level and adverse pregnancy outcomes has been reported in previous studies. Among these studies, the Hyperglycemia and Adverse Pregnancy Outcome (HAPO) Study has provided the most convincing data [13]. In pregnant women who had both an HbA1c level and 75-g, 2-h OGTT, both measurements were significantly associated with adverse pregnancy outcomes; however, the associations were more significant for the OGTT than the HbA1c level. Thus, the authors of the HAPO study concluded that the HbA1c level was not a useful alternative to an OGTT in pregnant women. However, more recent studies have linked the HbA1c level to adverse pregnancy outcomes using different cut-off points for the HbA1c level [14–16].

The objectives of this study were to determine the association between the mid-pregnancy HbA1c level and GDM diagnosed using a two-step approach with the Carpenter-Coustan criteria [17] and investigate whether the mid-pregnancy HbA1c level was associated with adverse pregnancy outcomes in women without overt diabetes and with positive 50-g, 1-h glucose challenge test (GCT) results (140 mg/dL or greater).

## Research design and methods

### Study participants

**Fig 1:**
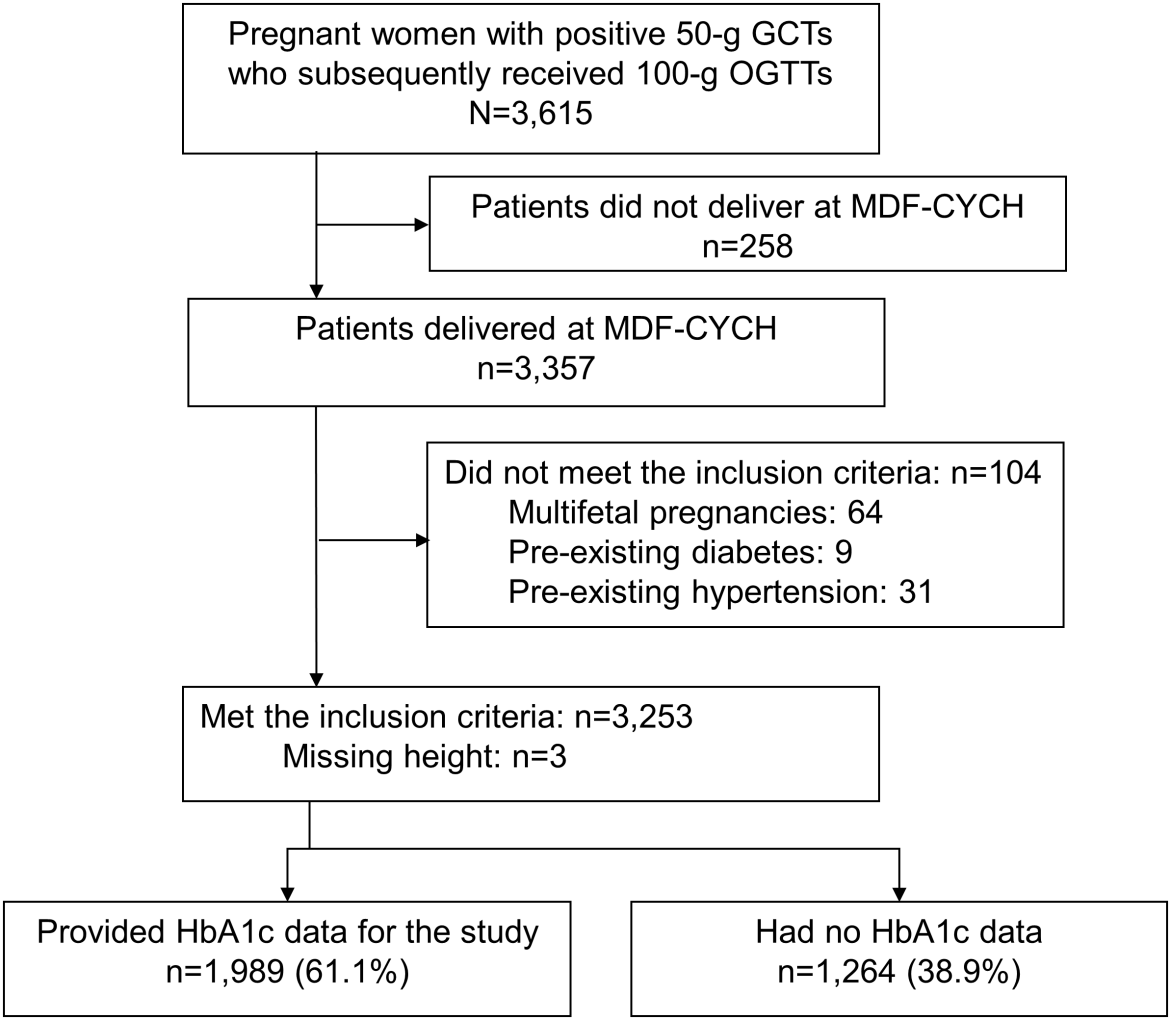
Enrollment of study subjects. GCT, glucose challenge test; OGTT, oral glucose tolerance test; DMF-CYCH, Ditmanson Medical Foundation Chia-Yi Christian Hospital; HbA1c, hemoglobin A1c.

This prospective study enrolled all pregnant women without overt diabetes and with positive 50-g, 1-h GCT results who subsequently underwent a 100-g, 3-h OGTT at the outpatient clinics of the Ditmanson Medical Foundation Chia-Yi Christian Hospital (DMF-CYCH) between March 2006 and September 2013. This study was approved by the Institutional Review Board (IRB) of the DMF-CYCH (CYCH IRB No: 100006). We collected data from medical records; thus, the committee agreed that the informed consent of each participant was not necessary. Data including nulliparous status, maternal age, body mass index (BMI), delivery year, and pregnancy outcomes were obtained from patient medical records. The study participants were provided with a two-step diagnostic approach for GDM, and an additional mid-pregnancy HbA1c test at the time the 100-g, 3-h OGTT was performed. Women who did not deliver their child at the DMF-CYCH, refused to participate in this study, or had multifetal pregnancies, pre-existing diabetes, and pre-existing hypertension were excluded. The enrollment of the study subjects is presented in Fig 1.

### Two-step diagnostic approach for GDM

**Fig 2:**
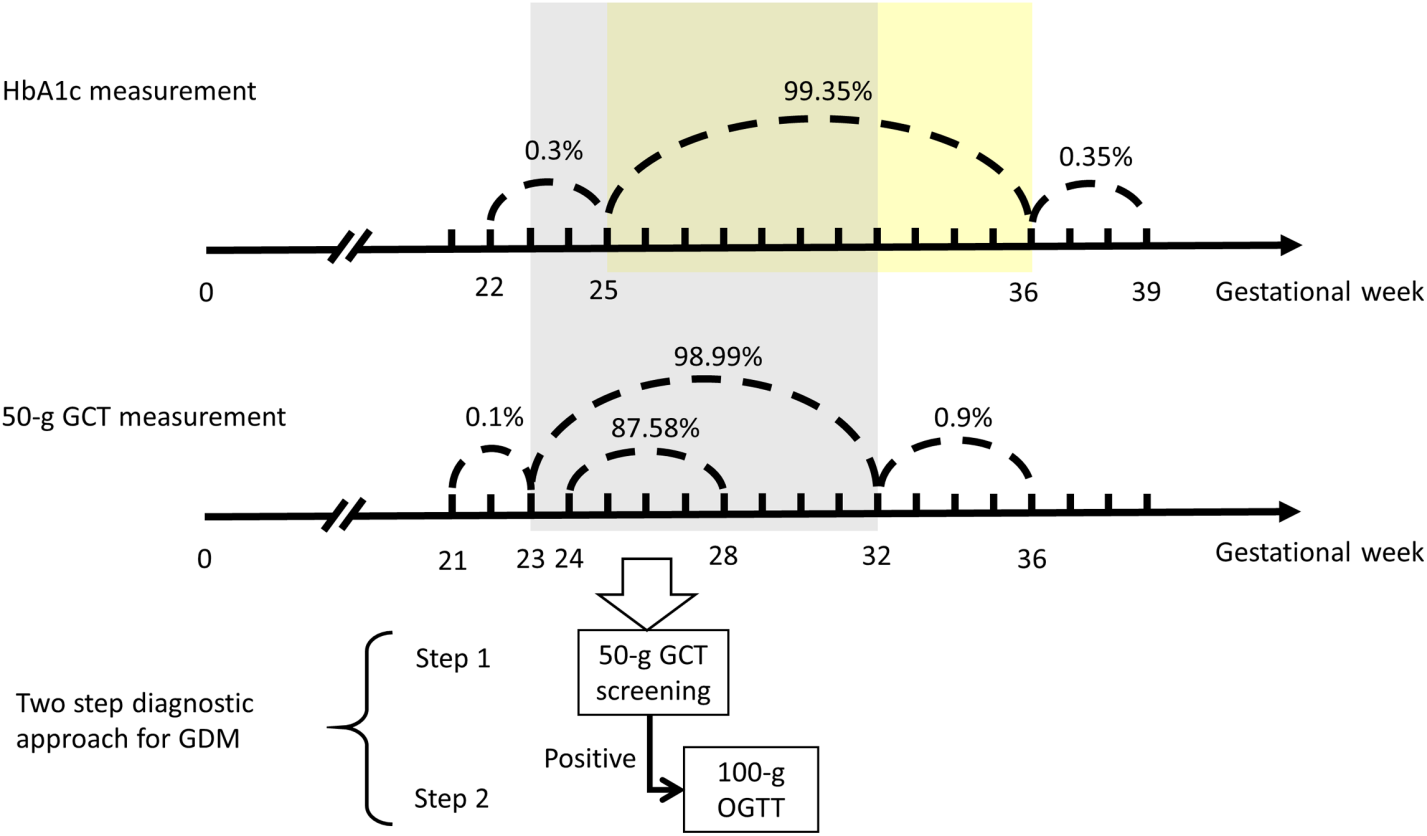
Gestational week of GDM screening using a two-step diagnostic approach and time frame for receiving the HbA1c test. GCT, glucose challenge test; OGTT, oral glucose tolerance test; GDM, gestational diabetes mellitus.

As a result of the health policy and National Health Insurance (NHI) coverage, most non-diabetic pregnant women in Taiwan were administered a 50-g, 1-h GCT at 24–28 weeks of gestation. The NHI provided 10 prenatal examinations by obstetrician gynecologists for pregnant women. Our study was based on the prenatal visit service of the NHI. If the GCT-measured levels were 140 mg/dL or greater (GCT-positive), then the women underwent a 100-g, 3-h OGTT. The OGTTs were arranged and performed in the morning outpatient clinic, and the patients fasted for at least 8 hours prior to the tests. GDM was diagnosed when the plasma glucose levels were considered abnormal according to the Carpenter-Coustan criteria, which indicated levels ≥95, 180, 155, and 140 mg/dL for the fasting, 1-hour, 2-hour and 3-hour plasma glucose tests, respectively [17]. Two or more abnormal glucose levels resulted in a GDM diagnosis. In this study, the venous plasma glucose levels were measured using the hexokinase-G6PDH method with a Hitachi 7170 automatic analyzer (Hitachi Co., Tokyo, Japan) in the DMF-CYCH central laboratory according to a standard clinical protocol. Fig 2 indicates the gestational week when the study subjects were screened for GDM using a two-step diagnostic approach and the time frame during which the study subjects received an HbA1c test.

### HbA1c measurement and classification

HbA1c was measured at the time the 100-g, 3-h OGTT was performed. For this test, blood was drawn in the morning following a fasting period of at least 8 hours. The HbA1c level was measured using the ion-exchange HPLC method with a Tosoh G8 automated analyzer (Tosoh Bioscience, Tokyo, Japan); the within-assay variation was 0.56%, and the between-assay variation was 1.15%. The normal range of this HbA1c level measurement was 4.0–6.0% according to the manufacturer. Based on a previous study that investigated the HbA1c level and disease outcomes, we calculated the pregnancy outcome rates via 0.5% increments of the HbA1c level (<4.5%, 4.5–4.9%, 5.0–5.4%, 5.5–5.9%, 6.0–6.4%, 6.5–6.9% and ≥7.0%) [18].

### Adverse pregnancy outcomes

Adverse pregnancy outcome data were collected, including caesarean section, prolonged labor, shoulder dystocia, third- or fourth-degree perineal laceration, postpartum hemorrhage, gestational hypertension or preeclampsia, preterm delivery (<37 weeks), fetal/neonatal death, admission to the neonatal intensive care unit, low birth weight (<2,500 g), macrosomia (>4,000 g), and Apgar scores <7 at 1 minute or 5 minutes. Caesarean sections as a result of prolonged labor, macrosomia, or cephalopelvic disproportion were included, whereas elective caesarean sections and caesarean sections scheduled because of a previous caesarean section, placenta previa, or malpositioning or malpresentation of the fetus were excluded. Gestational hypertension was defined as a systolic blood pressure ≥140 mmHg or a diastolic blood pressure ≥90 mmHg after 20 weeks of gestation in a woman with previously normal blood pressure and blood pressure levels that returned to normal postpartum. Preeclampsia was characterized by gestational hypertension and proteinuria (≥0.3 g/day or ≥1+ on a urine dipstick) with or without pathologic edema.

### Statistical analysis

Continuous variables are descriptively expressed as the mean ± standard deviation (SD) and were analyzed using analysis of variance (ANOVA); alternatively, they are expressed as the median (25th-75th) and were analyzed using non-parametric tests (the Kruskal-Wallis test or the Wilcoxon rank-sum test) when the data were not normally distributed. Proportions were determined for categorical variables using the Chi-squared test or Fisher’s exact test, as appropriate. To determine the association between the mid-pregnancy HbA1c level and GDM, a receiver operating characteristic (ROC) curve was constructed. To investigate whether the mid-pregnancy HbA1c level was significantly associated with the risk of adverse pregnancy outcomes, the Cochran-Armitage trend test was employed in the univariate analysis. Furthermore, multiple logistic regression models were used to calculate the odds ratios (ORs) and 95% confidence intervals (CIs) for the adverse pregnancy outcomes and were adjusted for nulliparous status, maternal age, BMI at delivery, and delivery year. *P* values for trends were based on the HbA1c category as a continuous scale. HbA1c was associated with GDM and many pregnancy complications examined in this study; thus, we further adjusted for it in an additional analysis. All statistical analyses were performed with SAS 9.2 (SAS Institute, Cary, NC, USA), and the ROC curve was generated using IBM SPSS Statistics Version 21. *P*<0.05 was considered statistically significant.

## Results

The study enrolled 3,615 pregnant women without overt diabetes and with positive 50-g, 1-h GCT results who subsequently underwent the 100-g, 3-h OGTT and delivered at DMF-CYCH during the study period. One hundred four women with multifetal pregnancies, pre-existing diabetes or pre-existing hypertension were excluded from this study. In addition, 1,264 patients (38.9%) refused to receive the HbA1c measurement. Thus, 1,989 (61.1%) pregnant women were included in the final analysis (Fig 1).

The baseline maternal characteristics of the study population are presented in S1 Table. The maternal age of the study population was 31.0 (28.0–34.4) years old. The nulliparous status was 50.5% (1,004/1,989). The pre-pregnancy BMI and BMI at delivery were 22.4 (20.0–24.8) and 26.8 (24.7–29.5), respectively. The incidence of gestational hypertension or preeclampsia was 5.0% (100/1,989).

To assess the difference between the study participants and non-participants, we performed an additional analysis of the potential selection bias. The results indicated that there were no differences in the maternal characteristics, glucose levels, or pregnancy outcomes between the HbA1c and non-HbA1c groups, except for the results for the 100-g, 1-h OGTT and the prolonged labor outcome (S1 Table). We also identified similar overall rates of GDM diagnosis in the patients who did versus did not undergo HbA1c monitoring (29.0% vs. 26.7%, respectively *P* = 0.16). Thus, there appeared to be no selection bias in the HbA1c group.

**Fig 3:**
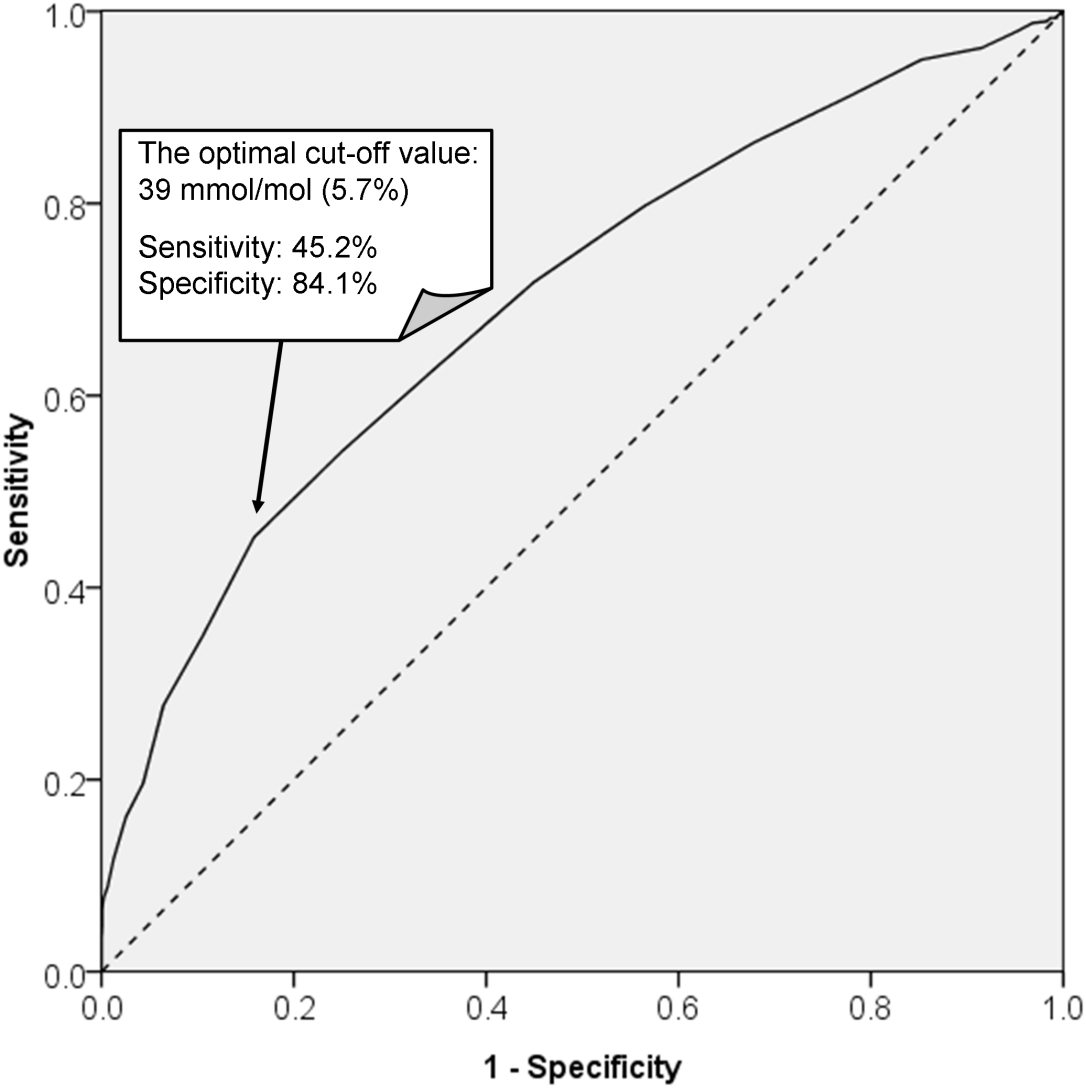
ROC curve indicates the sensitivity and specificity of HbA1c levels for detecting GDM. ROC, receiver operating characteristic; HbA1c, hemoglobin A1c; GDM, gestational diabetes mellitus.

An ROC curve (Fig 3) was generated to determine the sensitivity and specificity of the mid-pregnancy HbA1c level in the detection of GDM. The area under the ROC curve for HbA1c in the detection of GDM was 0.70 (95% CI 0.67–0.73). The optimal cut-off value, which maximized the sum of the sensitivity and specificity, was 5.7% (sensitivity of 45.2% and specificity of 84.1%) for GDM diagnosis.

Table 1 presents the rates of adverse pregnancy outcomes according to the mid-pregnancy HbA1c level. The rates of gestational hypertension or preeclampsia, preterm delivery, NICU admission, macrosomia, an Apgar score <7 at 5 minutes, and shoulder dystocia were significantly increased as the HbA1c levels increased. Only one woman had intrauterine fetal death, and her HbA1c level was 5.6%.

**Table 1:** Associations between HbA1c and pregnancy outcomes.

Outcome	No.	HbA1c category							Trend P^a^
	%	<4.5	4.5–4.9	5.0–5.4	5.5–5.9	6.0–6.4	6.5–6.9	≥7	
ALL case numbers	1,989	23	214	909	669	135	26	13	
GDM	576	4 (17.4)	25 (11.7)	188 (20.7)	247 (36.9)	75 (55.6)	25 (96.2)	12 (92.3)	<0.001
Caesarean section^b^	147	6 (26.1)	11 (5.1)	63 (6.9)	46 (6.9)	13 (9.6)	8 (30.8)	0 (0)	0.21
Prolonged labor	271	7 (30.4)	25 (11.7)	142 (15.6)	75 (11.2)	19 (14.1)	2 (7.7)	1 (7.7)	0.06
Postpartum hemorrhage	17	1 (4.4)	1 (0.5)	8 (0.9)	6 (0.9)	1 (0.7)	0 (0)	0 (0)	0.61
Gestational hypertension or preeclampsia	100	2 (8.7)	4 (1.9)	25 (2.8)	46 (6.9)	13 (9.6)	5 (19.2)	5 (38.5)	<0.001
Preterm delivery (<37 weeks)	163	2 (8.7)	11 (5.1)	62 (6.8)	62 (9.3)	21 (15.6)	2 (7.7)	3 (23.1)	<0.001
Admission to the NICU^c^	182	2 (8.7)	17 (7.9)	66 (7.3)	70 (10.5)	19 (14.1)	5 (19.2)	3 (23.1)	0.001
Low birth weight (<2,500 g)	139	2 (8.7)	14 (6.5)	51 (5.6)	59 (8.8)	10 (7.4)	1 (3.9)	2 (15.4)	0.13
Macrosomia (>4,000 g)	40	0 (0)	1 (0.5)	9 (1)	16 (2.4)	7 (5.2)	4 (15.4)	3 (23.1)	<0.001
Apgar score <7 at 1 minute	23	0 (0)	2 (0.9)	8 (0.9)	10 (1.5)	1 (0.7)	1 (3.9)	1 (7.7)	0.08
Apgar score <7 at 5 minutes	7	0 (0)	0 (0)	3 (0.3)	2 (0.3)	1 (0.7)	0 (0)	1 (7.7)	0.03
Vaginal delivery number	1,314	17	147	640	421	71	12	6	
Shoulder dystocia	14	0 (0)	0 (0)	4 (0.6)	5 (1.2)	5 (7)	0 (0)	0 (0)	0.001
Third- or fourth-degree perineal laceration	62	1 (5.9)	6 (4.1)	31 (4.8)	21 (5)	3 (4.2)	0 (0)	0 (0)	0.78

HbA1c, hemoglobin A1c; NICU, neonatal intensive care unit.

^a^ Data are presented as n (%), and trend *P* was calculated using the Cochran-Armitage trend test.

^b^ Caesarean section as a result of prolonged labor, macrosomia, or cephalopelvic disproportion, with the exclusion of elective caesarean sections and caesarean sections scheduled because of a previous cesarean section, placenta previa, or malposition or malpresentation of fetus.

^c^ Excludes fetal/neonatal deaths: only one woman had intrauterine fetal death.

Table 2 presents the trends and OR ranges. The multiple logistic regression analysis indicated that compared with the HbA1c category of 4.5–4.9%, higher HbA1c levels were significantly associated with increased risks of gestational hypertension or preeclampsia, preterm delivery, NICU admission, low birth weight, and macrosomia (OR ranges of 1.20–9.98, 1.31–5.16, 0.88–3.15, 0.89–4.10, and 2.22–27.86, respectively; all trend *P* values <0.05). The ORs for caesarean section, gestational hypertension or preeclampsia, preterm delivery, NICU admission, and low birth weight exhibited a J-shaped distribution. In the additional analysis (adjusted for both HbA1c and GDM), collinearity between the HbA1c level and GDM was identified; however, we elected to maintain both variables in the regression model to control for the possibility of bias, and the results were similar.

**Table 2:** Estimated odds ratios of pregnancy outcomes according to multiple regression analysis (n = 1,986).

Outcome	HbA1c category							Trend P^a^
	<4.5	4.5–4.9	5.0–5.4	5.5–5.9	6.0–6.4	6.5–6.9	≥7	
Caesarean section^b^	7.74	1	1.21	1.27	1.52	2.65^d^		0.73
	(2.34–25.57)		(0.61–2.37)	(0.63–2.56)	(0.62–3.73)	(0.88–8.00)		
Prolonged labor	3.44	1	1.42	1.03	1.38	0.69	0.66	0.17
	(1.28–9.24)		(0.90–2.24)	(0.63–1.69)	(0.71–2.67)	(0.15–3.17)	(0.08–5.39)	
Gestational hypertension or preeclampsia	4.14	1	1.2	2.51	2.79	3.34	9.98	0.001
	(0.70–24.59)		(0.41–3.51)	(0.87–7.22)	(0.84–9.20)	(0.76–14.73)	(2.01–49.52)	
Preterm delivery (<37 weeks)	1.67	1	1.31	1.76	3.01	1.31	5.16	0.003
	(0.35–8.07)		(0.68–2.55)	(0.90–3.45)	(1.36–6.65)	(0.26–6.50)	(1.19–22.30)	
Admission to the NICU^c^	1.06	1	0.88	1.29	1.71	2.3	3.15	0.005
	(0.23–4.90)		(0.51–1.54)	(0.73–2.28)	(0.83–3.53)	(0.73–7.20)	(0.76–12.98)	
Low birth weight (<2,500 g)	1.35	1	0.89	1.57	1.41	0.82	4.1	0.03
	(0.28–6.48)		(0.48–1.64)	(0.84–2.94)	(0.59–3.39)	(0.10–6.78)	(0.76–22.06)	
Macrosomia (>4,000 g)	1^d^		2.22	4.31	8.16	20.37	27.86	<0.001
			(0.28–17.70)	(0.56–33.20)	(0.95–69.87)	(1.99–208.87)	(2.40–323.25)	
Apgar score <7 at 1 minute	1^d^		1.09	1.63	0.67	2.93	8.03	0.23
			(0.23–5.23)	(0.34–7.80)	(0.06–8.08)	(0.22–39.98)	(0.60–107.16)	

HbA1c, hemoglobin A1c; NICU, neonatal intensive care unit. Data are presented as odds ratios (95% confidence interval) and adjusted for nulliparous status, maternal age, BMI at delivery, and delivery year in multiple logistic regressions.

^a^ P for trend based on the HbA1c category as a continuous scale.

^b^ Caesarean section as a result of prolonged labor, macrosomia, or cephalopelvic disproportion, with the exclusion of elective caesarean sections and caesarean sections scheduled because of a previous cesarean section, placenta previa, or malposition or malpresentation of fetus.

^c^ Excludes fetal/neonatal deaths: only one woman had intrauterine fetal death.

^d^ Combined HbA1c <4.5% and 4.5–4.9% as a result of no event occurrence in the HbA1c <4.5% group; combined HbA1c 6.5–6.9% and ≥7% as a result of no event occurrence in the HbA1c ≥7% group.

The associations between maternal characteristics and the HbA1c level are shown in S2 Table. An increasing maternal age and BMI at delivery significantly correlated with increasing HbA1c levels (*P*<0.001 for both). For the 48% (955/1,989) of women who provided their pre-pregnancy weight, we calculated the correlation between the pre-pregnancy BMI and BMI at delivery (data not shown). These two variables exhibited a strong positive correlation (Pearson’s correlation coefficient 0.88, *P*<0.001); therefore, we substituted the pre-pregnancy BMI for the BMI at delivery in the follow-up primary analysis.

## Discussion

In this study, we determined that the optimal cut-off point of the HbA1c level (with maximal sensitivity and specificity) to predict GDM was 5.7%. The area under the ROC curve of the HbA1c level for the detection of GDM was 0.70. However, the sensitivity was 45%, and the specificity was 84%. These findings confirmed the lack of adequate sensitivity and specificity in many previous studies [11,19–22]. In Osmundson and colleagues’ report, pregnant women with an HbA1c level of 5.7–6.4% were compared with an HbA1c level <5.7%. GDM was diagnosed by the International Association of Diabetes and Pregnancy Study Groups (IADPSG) criteria using a one-step 75-g, 2-h OGTT. The results indicated a first-trimester HbA1c level of 5.7–6.4% was associated with a low sensitivity (13%) and a 94% specificity in the prediction of GDM [21]. In a study by Renz and colleagues, GDM was diagnosed by the World Health Organization (WHO) 1999 and American Diabetes Association (ADA)/WHO 2013 criteria. An HbA1c level ≥5.8% exhibited 95% specificity but low sensitivity (26%) [20]. In another study, the OGTT and HbA1c tests were performed in 500 pregnant women at 24–28 weeks of gestation. The cut-off point of the HbA1c level was 5.3%, which had a sensitivity of 95.6% and a low specificity (52%) [19]. In 321 Korean women, the HbA1c level at a cut-off point of 5.05% exhibited 91% sensitivity and 62% specificity [22].

We further attempted to salvage a positive outlook on the use of mid-pregnancy HbA1c levels. A further objective was to determine whether a combination of maternal age, the 50-g, 1-h GCT and the mid-pregnancy HbA1c level would reduce the need for a subsequent 100-g, 3-h OGTT. However, the results indicated the algorithm could only prevent a very small proportion of patients with a positive screen (4%) from undergoing a full 100-g, 3-h OGTT (S1 Fig). Thus, we concluded that the mid-pregnancy HbA1c level could not replace a two-step diagnostic approach to identify GDM. However, a recent study reported that mid-pregnancy HbA1c may potentially reduce the number of OGTTs. In a study of 677 Nordic Caucasian women using the IADPSG criteria, approximately one-third of OGTTs could potentially be reduced using the mid-pregnancy HbA1c level with a sensitivity of 97% at weeks 32–36. Sixteen percent of the OGTTs could be avoided with a sensitivity of 96% [11]. It appears different criteria (the Carpenter-Coustan criteria using a two-step diagnostic approach vs. the IADPSG criteria using a one-step 75-g, 2-h OGTT) for GDM diagnosis affect the results. Further comparison studies are required.

Our findings demonstrated that the mid-pregnancy HbA1c level was associated with various adverse pregnancy outcomes in a continuous fashion. These outcomes included gestational hypertension or preeclampsia, preterm delivery, NICU admission, low birth weight, and macrosomia. The results provided supporting evidence for recent reports that the HbA1c level during pregnancy was associated with adverse pregnancy outcomes [14–16]. Thus, the mid-pregnancy HbA1c level may be used as a prognostic biomarker for adverse pregnancy outcomes.

In addition, compared with the women with HbA1c levels of 4.5–4.9% (reference group), the women with lower or higher HbA1c levels (<4.5% and ≥6.0%) had a higher risk of adverse pregnancy outcomes, which demonstrated a J-shaped curve for risk. Similar J-shaped relationships have been identified for HbA1c levels and cardiovascular, cancer and all-cause mortality in patients with diabetes in the Ludwigshafen Risk and Cardiovascular Health study [23]. For adults without overt diabetes, a J-shaped relationship was also identified between the HbA1c levels and all-cause mortality in the Atherosclerosis Risk in Communities (ARIC) study and in a New Zealand linkage study [24,25]. However, there have been limited studies regarding this relationship in pregnant women. We hypothesized that low HbA1c levels measured at GDM diagnosis may potentially reflect a chronic, consuming physiopathological condition, which may lead to adverse pregnancy outcomes. Additional studies are required to confirm these results and determine the potential mechanisms that may underlie this association [26,27].

The strength of the study was the assessment of the clinical usefulness of a mid-pregnancy HbA1c measurement as a replacement for the OGTT in pregnancy using a relatively large population over a 7-year period. In Taiwan, the NHI provided 10 prenatal examinations by obstetrician gynecologists for pregnant women. Our study was based on the prenatal visit service of the NHI, which may refine the HbA1c measurement in this study and the diagnosis of GDM. Nevertheless, because of the single-center non-randomized design, we should be cautious regarding the generalizability. Additional, large-scale, multi-center, randomized control design studies are required.

## Conclusions

The mid-pregnancy HbA1c level was associated with various adverse pregnancy outcomes in high-risk Taiwanese women. However, it lacked adequate sensitivity and specificity to replace a two-step diagnostic approach for GDM. The current study was a single-center prospective study; thus, additional, randomized control design studies are required.

## Supporting information

S1 Fig Proposed algorithm for avoiding 100-g OGTTs. GCT, glucose challenge test; HbA1c, hemoglobin A1c; GDM, gestational diabetes mellitus; OGTT, oral glucose tolerance test. * Predicts GDM: 94.9% (37/39); Age: 24.8–43.7 years; Glucose level from the GCT: 140–323 mg/dL. † Predicts GDM: 0% (27/27); Glucose level from the GCT: 142–182 mg/dL. ‡ Predicts GDM: 100% (14/14).
(TIF)

S1 File The analysis data. (XLS)

S1 Table Differences in the maternal characteristics, glucose levels, and pregnancy outcomes between the HbA1c and non-HbA1c groups. HbA1c, hemoglobin A1c; BMI, body mass index; GCT, glucose challenge test; OGTT, oral glucose tolerance test; GDM, gestational diabetes mellitus; NICU, neonatal intensive care unit. Continuous variables are presented as the median (25th-75th) and were analyzed using the Wilcoxon rank sum test. Categorical variables are presented as n (%) and were analyzed using the Chi-squared test or Fisher’s exact test, as appropriate. ^a^ Forty-eight percent (955/1,989) of cases provided their pre-pregnancy weight. ^b^ Caesarean as a result of prolonged labor, macrosomia, or cephalopelvic disproportion, with the exclusion of elective caesarean sections and caesarean sections scheduled because of a previous cesarean section, placenta previa, and malposition or malpresentation of fetus. ^c^ Only includes vaginal deliveries. ^d^ Excludes fetal/neonatal death.
(DOC)

S2 Table Associations between maternal characteristics and HbA1c. HbA1c, hemoglobin A1c; BMI, body mass index. Continuous variables are presented as the mean ± SD or median (25th-75th) and were analyzed using analysis of variance (ANOVA) or the Kruskal-Wallis test, as appropriate. Categorical variables are presented as n (%) and were analyzed using the Chi-squared test. ^a^ Forty-eight percent (955/1,989) of cases provided their pre-pregnancy weight.
(DOC)

## References

[1] McFarland KF, Murtiashaw M, Baynes JW. Clinical value of glycosylated serum protein and glycosylated hemoglobin levels in the diagnosis of gestational diabetes mellitus. Obstet Gynecol. 1984;64(4):516–8. Epub 1984/10/01. 6483299

[2] Cousins L, Dattel BJ, Hollingsworth DR, Zettner A. Glycosylated hemoglobin as a screening test for carbohydrate intolerance in pregnancy. Am J Obstet Gynecol. 1984;150(5 Pt 1):455–60. Epub 1984/11/01. 6496578 10.1016/s0002-9378(84)90420-4

[3] Artal R, Mosley GM, Dorey FJ. Glycohemoglobin as a screening test for gestational diabetes. Am J Obstet Gynecol. 1984;148(4):412–4. Epub 1984/02/15. 6695998 10.1016/0002-9378(84)90717-8

[4] Rajput R, Yogesh Y, Rajput M, Nanda S. Utility of HbA1c for diagnosis of gestational diabetes mellitus. Diabetes Res Clin Pract. 2012;98(1):104–7. Epub 2012/03/30. 10.1016/j.diabres.2012.02.018 22456454

[5] Moses RG. HbA1c and the diagnosis of gestational diabetes mellitus—a test whose time has not yet come. Diabetes Res Clin Pract. 2012;98(1):3–4. Epub 2012/06/22. 10.1016/j.diabres.2012.05.016 22717498

[6] Agarwal MM, Dhatt GS, Punnose J, Koster G. Gestational diabetes: a reappraisal of HBA1c as a screening test. Acta Obstet Gynecol Scand. 2005;84(12):1159–63. Epub 2005/11/25. 10.1111/j.0001-6349.2005.00650.x 16305701

[7] Odsaeter IH, Asberg A, Vanky E, Carlsen SM. HbA1c as screening for gestational diabetes mellitus in women with polycystic ovary syndrome. BMC Endocr Disord. 2015;15:38 Epub 2015/08/08. 10.1186/s12902-015-0039-9 26245653 PMC4527320

[8] Garner LA, Miller E, Katon J. First-Trimester A1C as a Tool to Predict the Development of Gestational Diabetes in High-Risk Women. Obstet Gynecol. 2014;123:52S.

[9] Phelps RL, Honig GR, Green D, Metzger BE, Frederiksen MC, Freinkel N. Biphasic changes in hemoglobin A1c concentrations during normal human pregnancy. Am J Obstet Gynecol. 1983;147(6):651–3. Epub 1983/11/15. 6605685 10.1016/0002-9378(83)90443-x

[10] Hughes RC, Rowan J, Florkowski CM. Is There a Role for HbA1c in Pregnancy? Curr Diab Rep. 2016;16(1):5 Epub 2016/01/08. 10.1007/s11892-015-0698-y 26739347

[11] Odsaeter IH, Asberg A, Vanky E, Morkved S, Stafne SN, Salvesen KA, et al Hemoglobin A1c as screening for gestational diabetes mellitus in Nordic Caucasian women. Diabetol Metab Syndr. 2016;8:43 Epub 2016/07/28. 10.1186/s13098-016-0168-y 27453735 PMC4957925

[12] Claesson R, Ignell C, Shaat N, Berntorp K. HbA1c as a predictor of diabetes after gestational diabetes mellitus. Prim Care Diabetes. 2017;11(1):46–51. Epub 2016/10/04. 10.1016/j.pcd.2016.09.002 27692850

[13] Lowe LP, Metzger BE, Dyer AR, Lowe J, McCance DR, Lappin TR, et al Hyperglycemia and Adverse Pregnancy Outcome (HAPO) Study: associations of maternal A1C and glucose with pregnancy outcomes. Diabetes Care. 2012;35(3):574–80. Epub 2012/02/04. 10.2337/dc11-1687 22301123 PMC3322718

[14] Ye M, Liu Y, Cao X, Yao F, Liu B, Li Y, et al The utility of HbA1c for screening gestational diabetes mellitus and its relationship with adverse pregnancy outcomes. Diabetes Res Clin Pract. 2016;114:43–9. Epub 2016/04/23. 10.1016/j.diabres.2016.02.007 27103368

[15] Hughes RC, Moore MP, Gullam JE, Mohamed K, Rowan J. An early pregnancy HbA1c >/ = 5.9% (41 mmol/mol) is optimal for detecting diabetes and identifies women at increased risk of adverse pregnancy outcomes. Diabetes Care. 2014;37(11):2953–9. Epub 2014/09/06. 10.2337/dc14-1312 25190675

[16] Anaka O, Houlihan C, Beim R, Ranzini AC. Does First-Trimester Hemoglobin A1C Predict Gestational Diabetes and Fetal Outcome? Obstet Gynecol. 2014;123:38S–9S. PubMed-201405001-00079.

[17] Carpenter MW, Coustan DR. Criteria for screening tests for gestational diabetes. Am J Obstet Gynecol. 1982;144(7):768–73. Epub 1982/12/01. 7148898 10.1016/0002-9378(82)90349-0

[18] Chamnan P, Simmons RK, Khaw KT, Wareham NJ, Griffin SJ. Change in HbA1c over 3 years does not improve the prediction of cardiovascular disease over and above HbA1c measured at a single time point. Diabetologia. 2013;56(5):1004–11. Epub 2013/02/14. 10.1007/s00125-013-2854-8 23404444 PMC3776254

[19] Soumya S, Rohilla M, Chopra S, Dutta S, Bhansali A, Parthan G, et al HbA1c: A Useful Screening Test for Gestational Diabetes Mellitus. Diabetes Technol Ther. 2015;17(12):899–904. Epub 2015/10/27. 10.1089/dia.2015.0041 26496534

[20] Renz PB, Cavagnolli G, Weinert LS, Silveiro SP, Camargo JL. HbA1c Test as a Tool in the Diagnosis of Gestational Diabetes Mellitus. PLoS One. 2015;10(8):e0135989 Epub 2015/08/21. 10.1371/journal.pone.0135989 26292213 PMC4546239

[21] Osmundson SS, Zhao BS, Kunz L, Wang E, Popat R, Nimbal VC, et al First Trimester Hemoglobin A1c Prediction of Gestational Diabetes. Am J Perinatol. 2016;33(10):977–82. Epub 2016/04/28. 10.1055/s-0036-1581055 27120479

[22] Kwon SS, Kwon JY, Park YW, Kim YH, Lim JB. HbA1c for diagnosis and prognosis of gestational diabetes mellitus. Diabetes Res Clin Pract. 2015;110(1):38–43. Epub 2015/09/08. 10.1016/j.diabres.2015.07.014 26344325

[23] Silbernagel G, Grammer TB, Winkelmann BR, Boehm BO, März W. Glycated hemoglobin predicts all-cause, cardiovascular, and cancer mortality in people without a history of diabetes undergoing coronary angiography. Diabetes Care. 2011;34(6):1355–61. 10.2337/dc10-2010 21515847 PMC3114349

[24] Selvin E, Steffes MW, Zhu H, Matsushita K, Wagenknecht L, Pankow J, et al Glycated hemoglobin, diabetes, and cardiovascular risk in nondiabetic adults. N Engl J Med. 2010;362(9):800–11. 10.1056/NEJMoa0908359 20200384 PMC2872990

[25] Brewer N, Wright CS, Travier N, Cunningham CW, Hornell J, Pearce N, et al A New Zealand linkage study examining the associations between A1C concentration and mortality. Diabetes Care. 2008;31(6):1144–9. 10.2337/dc07-2374 18299440

[26] Aggarwal V, Schneider AL, Selvin E. Low Hemoglobin A1c in Nondiabetic Adults An elevated risk state? Diabetes Care. 2012;35(10):2055–60. 10.2337/dc11-2531 22855733 PMC3447844

[27] Di Angelantonio E, Gao P, Khan H, Butterworth AS, Wormser D, Kaptoge S, et al Glycated hemoglobin measurement and prediction of cardiovascular disease. JAMA. 2014;311(12):1225–33. 10.1001/jama.2014.1873 24668104 PMC4386007

