# Supplementary figures and images for: Associations of mid-pregnancy HbA1c with gestational diabetes and risk of adverse pregnancy outcomes in high-risk Taiwanese women
Source: PLoS One. 2017 May 15;12(5):e0177563. doi: 10.1371/journal.pone.0177563 (PMC5432166; doi:10.1371/journal.pone.0177563)

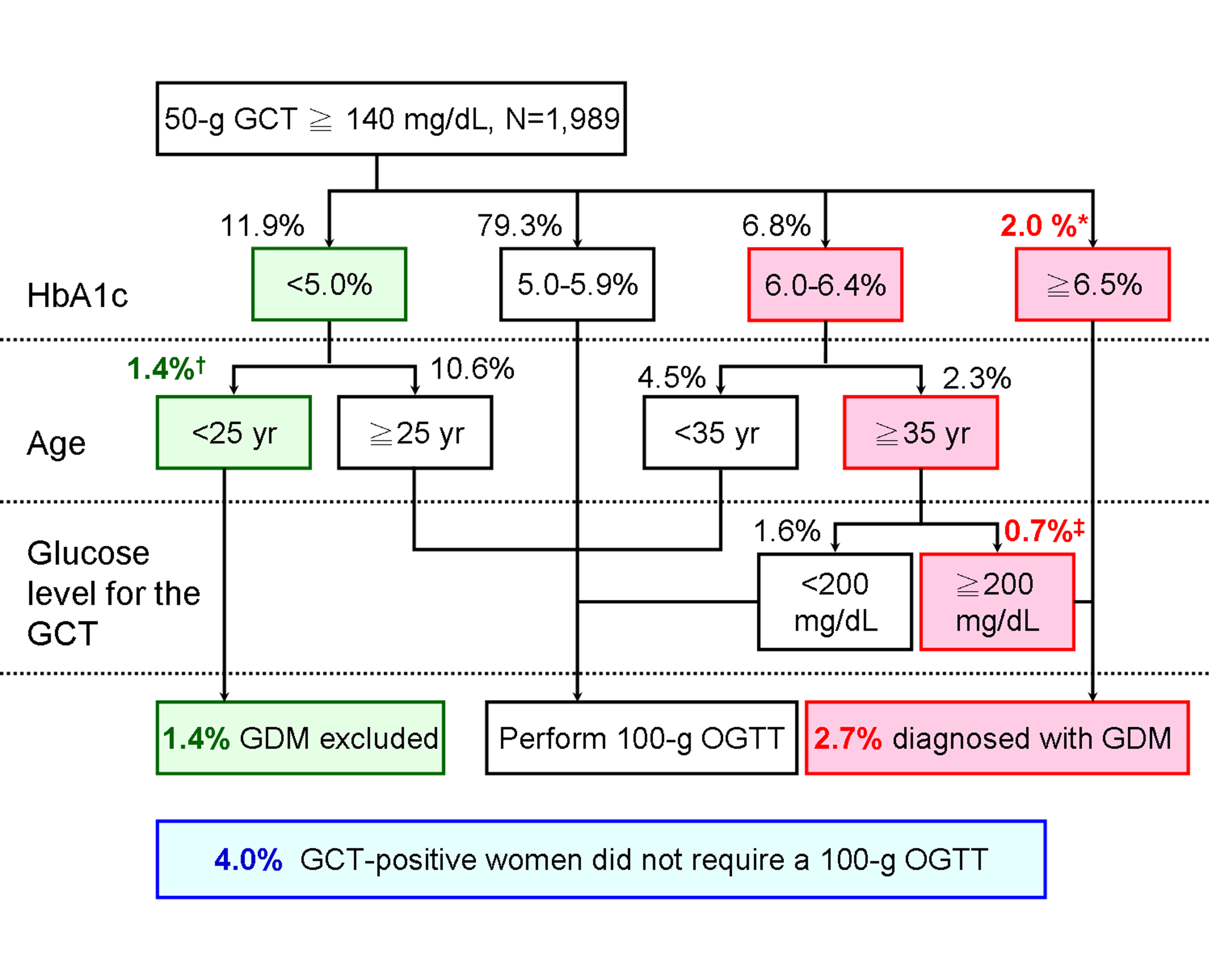

Supplement: S1 Fig — GCT, glucose challenge test; HbA1c, hemoglobin A1c; GDM, gestational diabetes mellitus; OGTT, oral glucose tolerance test. * Predicts GDM: 94.9% (37/39); Age: 24.8–43.7 years; Glucose level from the GCT: 140–323 mg/dL. † Predicts GDM: 0% (27/27); Glucose level from the GCT: 142–182 mg/dL. ‡ Predicts GDM: 100% (14/14). (TIF) [file pone.0177563.s001.tif]
