# Supplementary material for: Associations of mid-pregnancy HbA1c with gestational diabetes and risk of adverse pregnancy outcomes in high-risk Taiwanese women
Source: PLoS One. 2017 May 15;12(5):e0177563. doi: 10.1371/journal.pone.0177563 (PMC5432166; doi:10.1371/journal.pone.0177563)
Supplement: S1 Table — HbA1c, hemoglobin A1c; BMI, body mass index; GCT, glucose challenge test; OGTT, oral glucose tolerance test; GDM, gestational diabetes mellitus; NICU, neonatal intensive care unit. Continuous variables are presented as the median (25th-75th) and were analyzed using the Wilcoxon rank sum test. Categorical variables are presented as n (%) and were analyzed using the Chi-squared test or Fisher’s exact test, as appropriate. a Forty-eight percent (955/1,989) of cases provided their pre-pregnancy weight. b Caesarean as a result of prolonged labor, macrosomia, or cephalopelvic disproportion, with the exclusion of elective caesarean sections and caesarean sections scheduled because of a previous cesarean section, placenta previa, and malposition or malpresentation of fetus. c Only includes vaginal deliveries. d Excludes fetal/neonatal death. (DOC) [file pone.0177563.s003.doc]

**Supplemental Table 1. Differences in the maternal characteristics, glucose levels, and pregnancy outcomes between the HbA1c and non-HbA1c groups**.

| Variable | HbA1c | | P |
| --- | --- | --- | --- |
|  | Received (n=1,989) | None (n=1,264) |  |
| Maternal characteristics |  |  |  |
| Nulliparous status | 1,004 (50.5) | 643 (50.9) | 0.83 |
| Maternal age (yrs) | 31.0 (28.0-34.4) | 30.9 (28.2-34.1) | 0.84 |
| Pre-pregnancy BMI (kg/m^2^)^a^ | 22.4 (20.0-24.8) | 21.8 (20.0-24.4) | 0.12 |
| BMI at delivery (kg/m^2^) | 26.8 (24.7-29.5) | 27.0 (24.8-29.5) | 0.71 |
| Weight gain during pregnancy (kg) ^a^ | 11.4 (8.9-14.5) | 12.0 (9.4-14.8) | 0.05 |
| Glucose levels (mg/dL) |  |  |  |
| 50-g GCT positive | 157 (149-171) | 157 (148-171) | 0.31 |
| 100-g OGTT fasting | 83 (77-88) | 82 (78-88) | 0.71 |
| 100-g OGTT 1 h | 162 (143-183) | 159 (140.5-180) | 0.04 |
| 100-g OGTT 2 h | 144 (124-164) | 141 (123-162) | 0.08 |
| 100-g OGTT 3 h | 115 (97-133) | 115 (99-132.5) | 0.95 |
| GDM rate | 576 (29.0) | 337 (26.7) | 0.16 |
| Outcomes |  |  |  |
| Caesarean section^b^ | 147 (7.4) | 111 (8.8) | 0.15 |
| Prolonged labor | 271 (13.6) | 212 (16.8) | 0.01 |
| Shoulder dystocia^c^ | 14 (1.1) | 13 (1.7) | 0.20 |
| Third/fourth-degree perineal laceration^‡^ | 62 (4.7) | 44 (5.9) | 0.25 |
| Postpartum hemorrhage | 17 (0.9) | 12 (1.0) | 0.78 |
| Gestational hypertension or preeclampsia | 100 (5.0) | 75 (5.9) | 0.26 |
| Preterm delivery (<37 weeks) | 163 (8.2) | 95 (7.5) | 0.48 |
| Fetal/neonatal death | 1 (0.05) | 1 (0.08) | - |
| Admission to NICU^d^ | 182 (9.2) | 104 (8.2) | 0.37 |
| Low birth weight (<2,500 g) | 139 (7.0) | 72 (5.7) | 0.14 |
| Macrosomia (>4,000 g) | 40 (2.0) | 28 (2.2) | 0.69 |
| Apgar score <7 at 1 minute | 23 (1.2) | 16 (1.3) | 0.78 |
| Apgar score <7 at 5 minutes | 7 (0.4) | 4 (0.3) | 1.00 |

HbA1c, hemoglobin A1c; BMI, body mass index; GCT, glucose challenge test; OGTT, oral glucose tolerance test; GDM, gestational diabetes mellitus; NICU, neonatal intensive care unit.

Continuous variables are presented as the median (25th-75th) and were analyzed using the Wilcoxon rank sum test. Categorical variables are presented as n (%) and were analyzed using the Chi-squared test or Fisher’s exact test, as appropriate.

^a^Forty-eight percent (955/1,989) of cases provided their pre-pregnancy weight.

^b^Caesarean as a result of prolonged labor, macrosomia, or cephalopelvic disproportion, with the exclusion of elective caesarean sections and caesarean sections scheduled because of a previous cesarean section, placenta previa, and malposition or malpresentation of fetus.

^c^Only includes vaginal deliveries.

^d^Excludes fetal/neonatal death
