# Supplementary material for: Associations of mid-pregnancy HbA1c with gestational diabetes and risk of adverse pregnancy outcomes in high-risk Taiwanese women
Source: PLoS One. 2017 May 15;12(5):e0177563. doi: 10.1371/journal.pone.0177563 (PMC5432166; doi:10.1371/journal.pone.0177563)
Supplement: S2 Table — HbA1c, hemoglobin A1c; BMI, body mass index. Continuous variables are presented as the mean ± SD or median (25th-75th) and were analyzed using analysis of variance (ANOVA) or the Kruskal-Wallis test, as appropriate. Categorical variables are presented as n (%) and were analyzed using the Chi-squared test. a Forty-eight percent (955/1,989) of cases provided their pre-pregnancy weight. (DOC) [file pone.0177563.s004.doc]

**Supplemental Table 2. Associations between maternal characteristics and HbA1c.**

| Maternal characteristics | HbA1c category (%) | | | | | | | P |
| --- | --- | --- | --- | --- | --- | --- | --- | --- |
|  | <4.5 | 4.5-4.9 | 5.0-5.4 | 5.5-5.9 | 6.0-6.4 | 6.5-6.9 | ≥7 |  |
| Number | 23 | 214 | 909 | 669 | 135 | 26 | 13 |  |
| Nulliparous status | 12 (52.2) | 110 (51.4) | 498 (54.8) | 301 (45.0) | 61 (45.2) | 15 (57.7) | 7 (53.9) | 0.009 |
| Maternal age (yrs) | 31.2±5.4 | 30.3±4.5 | 30.7±4.4 | 31.7±4.5 | 32.6±5.3 | 32.8±5.1 | 30.7±4.8 | <0.001 |
| BMI at delivery (kg/m^2^) | 26.6  (24.5-29.5) | 25.6  (23.7-27.8) | 26.2  (24.3-28.7) | 27.6  (25.3-30.4) | 29.3  (27.3-32.0) | 32.4  (30.2-37.0) | 34.4  (31.8-35.0) | <0.001 |
| Number^a^ | 11 | 124 | 446 | 301 | 54 | 13 | 6 |  |
| Pre-pregnancy BMI (kg/m^2^) | 21.2  (19.0-22.0) | 20.2  (18.8-22.5) | 21.6  (19.7-23.9) | 23.4  (21.6-26.2) | 24.7  (22.6-27.3) | 27.2  (24.1-31.3) | 30.6  (28.4-32.7) | <0.001 |
| Weight gain during pregnancy (kg) | 11.8  (9.8-18.6) | 12.1  (9.5-14.85) | 11.4  (9.2-14.8) | 11  (8.2-14) | 11.6  (8.7-14.7) | 14.0  (9.9-15.4) | 6.5  (4.9-11.2) | 0.06 |

HbA1c, hemoglobin A1c; BMI, body mass index.

Continuous variables are presented as the mean ± SD or median (25th-75th) and were analyzed using analysis of variance (ANOVA) or the Kruskal-Wallis test, as appropriate. Categorical variables are presented as n (%) and were analyzed using the Chi-squared test.

^a^Forty-eight percent (955/1,989) of cases provided their pre-pregnancy weight.
